# Supplementary material for: TFEB regulates sulfur amino acid and coenzyme A metabolism to support hepatic metabolic adaptation and redox homeostasis
Source: Nat Commun. 2022 Sep 28;13:5696. doi: 10.1038/s41467-022-33465-9 (PMC9519740; doi:10.1038/s41467-022-33465-9)
Supplement: Supplementary file 1 — Supplementary Information [file 41467_2022_33465_MOESM1_ESM.pdf]

Supplementary Information

TFEB regulates sulfur amino acid and coenzyme A metabolism to support hepatic metabolic adaptation and redox homeostasis

David Matye et al.

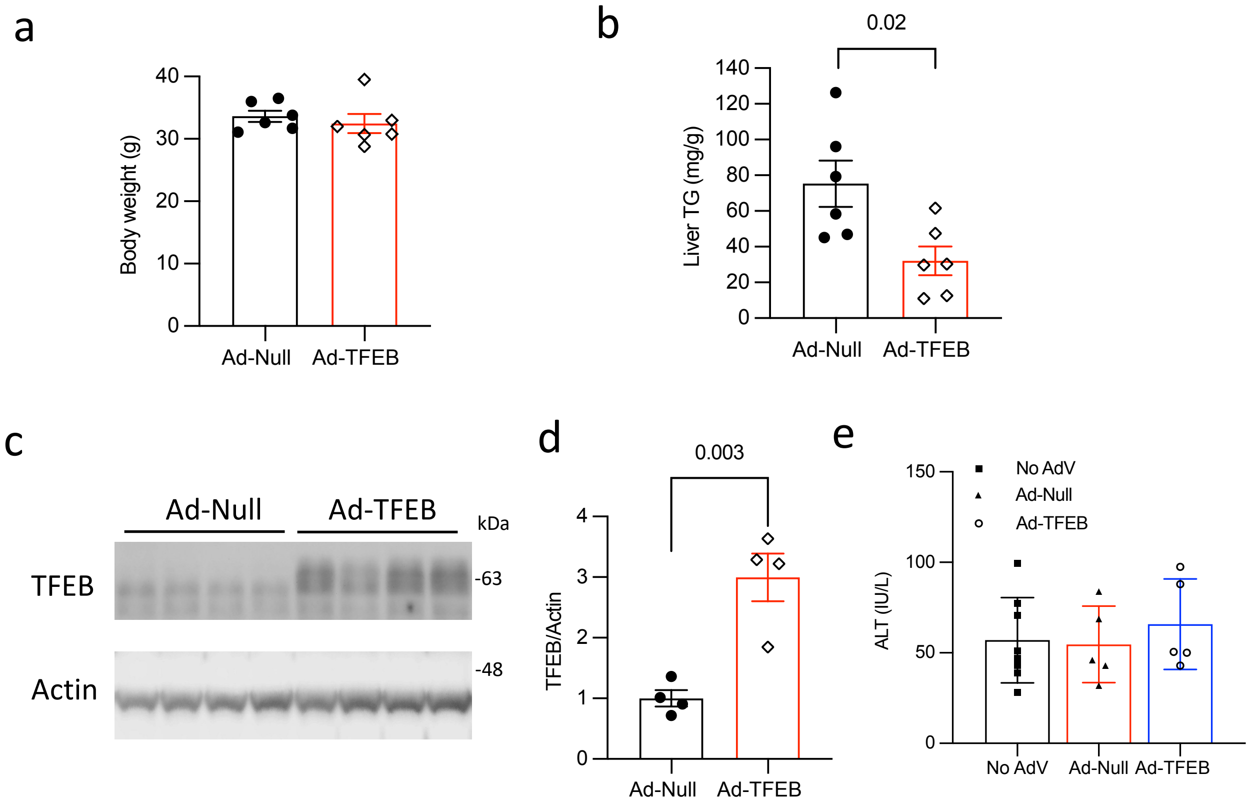

Supplementary Figure 1. Liver TFEB overexpression attenuates hepatic steatosis in 6 weeks WD-fed mice. a, b. Male 10 weeks old C57BL/6J mice were fed Western diet (WD) for 3 weeks, and then injected with Ad-Null or Ad-TFEB at $5X10^8$ pfu/mouse. These mice were fed WD for additional 3 weeks and euthanized. Body weight (a) and hepatic triglycerides (TG) (b) were measured at the end of the 6-week study. (n=6). **c, d, e.** Male 10 weeks old C57BL/6J mice were intravenously injected with Ad-Null or Ad-TFEB at $5X10^8$ pfu/mouse. Mice were fed chow diet for 2 weeks. **c.** Liver transcription factor EB (TFEB) overexpression is confirmed by Western blotting after 2 weeks. Each band represents an individual mouse sample. **d.** Densitometry was performed with ImageJ software. TFEB intensity is normalized to Actin intensity. **e.** Plasma alanine aminotransferase (ALT) at 2 weeks post injection (n=8 for No AdV; n=5 for Ad-Null and Ad-TFEB). Results are mean ± SEM. Unpaired 2-tailed t-test is used for b and d. Source data for a, b, c, d, e is provided as a Source Data file.

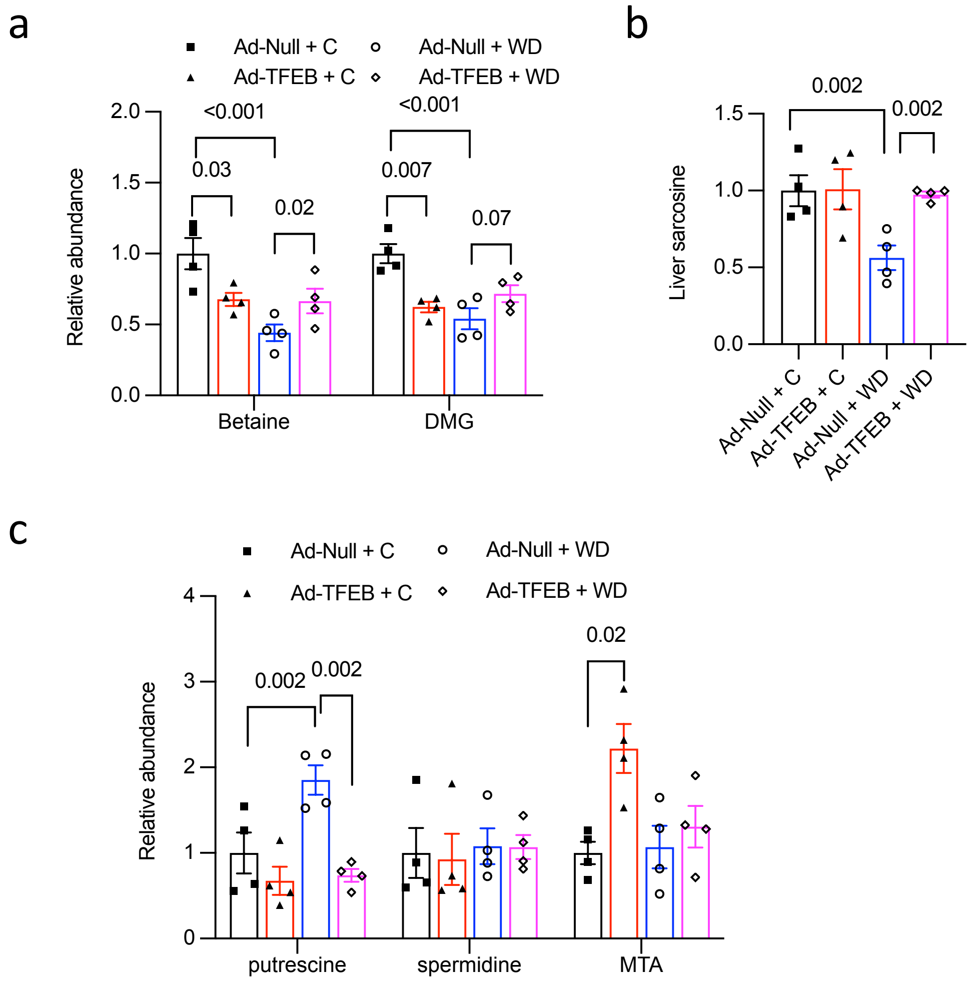

Supplementary Figure 2. TFEB effect on hepatic metabolic pathways in mice. a, b, c. Male 10 weeks old C57BL/6J mice were intravenously injected with Ad-Null or Ad-TFEB at $5X10^8$ pfu/mouse. One week later, mice were fed chow (C) or Western diet (WD) for one additional week. Relative abundance of liver metabolites are shown with control arbitrarily set as 1. All results are mean ± SEM (n=4). Detailed statistical analysis for a, b, c is described under Metabolomics, statistical and bioinformatics analysis in the Methods section. A p value < 0.05 is considered statistically significant. DMG: dimethylglycine; MTA:5'-methylthioadenosine. Source data for a-c is provided in the Source Data file.

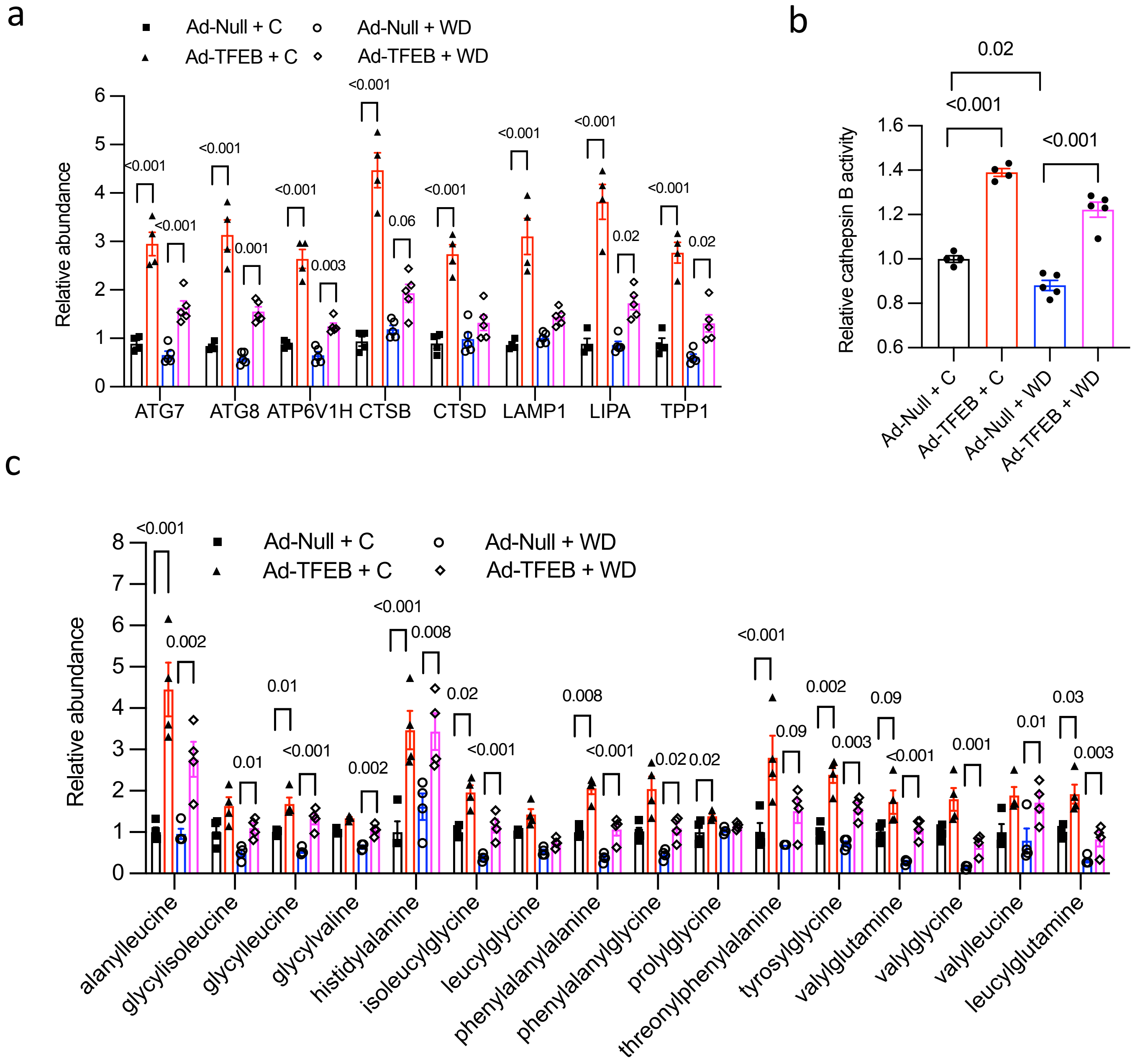

Supplementary Figure 3. TFEB induced hepatic autophagy and lysosome activity. Male 10 weeks old C57BL/6J mice were intravenously injected with Ad-Null or Ad-TFEB at 5X10^{8} pfu/mouse. One week later, mice were fed chow (C) or Western diet (WD) for one additional week. Mice were fasted for 6 h and euthanized. **a.** Relative liver mRNA expression (n=4 for chow condition, n=5 for WD condition). ATG7: Autophagy-related gene 7; ATG8: Autophagy-related gene 8; ATP6V1H: ATPase H^{+} Transporting V1 Subunit H; CTSB: cathepsin B; CTSD: cathepsin D; LAMP1: lysosomal associated membrane protein 1; LIPA: lipase A, lysosomal acid type; TPP1:Tripeptidyl Peptidase 1. **b.** Relative liver cathepsin B activity (n=4 for chow condition, n=5 for WD condition). **c.** Relative abundance of liver metabolites are shown with control arbitrarily set as 1 (n=4). Detailed statistical analysis of c is described under Metabolomics, statistical and bioinformatics analysis in the Methods section. Two-way ANOVA and Tukey post hoc test is used for a and b. A p value < 0.05 is considered statistically significant. Source data for a, b, c is provided as a Source Data file.

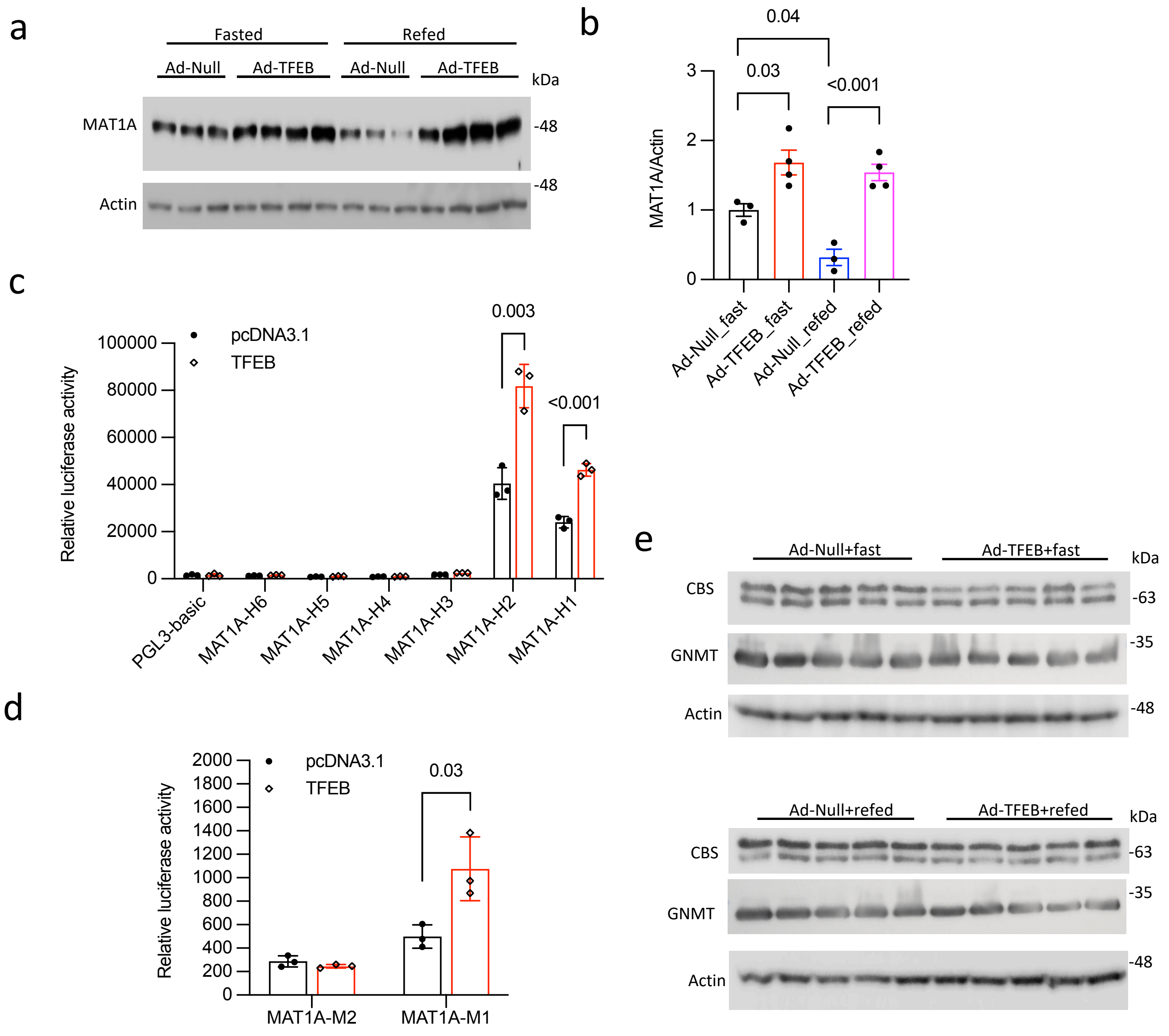

Supplementary Figure 4. TFEB induces hepatic MAT1A expression. Male 10 weeks old C57BL/6J mice were intravenously injected with Ad-Null or Ad-TFEB at 5X10^8 pfu/mouse. After 2 weeks, mice were either fasted for 22 h or fasted for 16 h and then refed with chow for 6 h. **a, b.** Liver methionine adenosyltransferase 1A (MAT1A) protein. Relative MAT1A band intensity normalized to Actin is shown in b. ImageJ software was used to measure band intensity. Each band represents an individual mouse sample. **c, d.** Luciferase reporter constructs (0.2 µg/well) containing human (c) or mouse (d) MAT1A promoter fragments as shown in Supplemental Table 1 and TFEB expression plasmid (0.1 µg/well) were transfected in triplicates in AML12 cells for 48h. Luciferase activity was normalized to β-galactosidase activity. A representative of 2-3 independent experiments. **e.** Liver Cystathionine β-Synthase (CBS) and Glycine N-Methyltransferase (GNMT) protein. Each band represents an individual mouse sample. Result in b is expressed as mean ± SEM and results in c and d are expressed as mean ± SD. A p value < 0.05 is considered statistically significant (2-way ANOVA and Tukey post hoc test for b and Unpaired 2-tailed t-test for c and d). Source data for a-e is provided as a Source Data file.

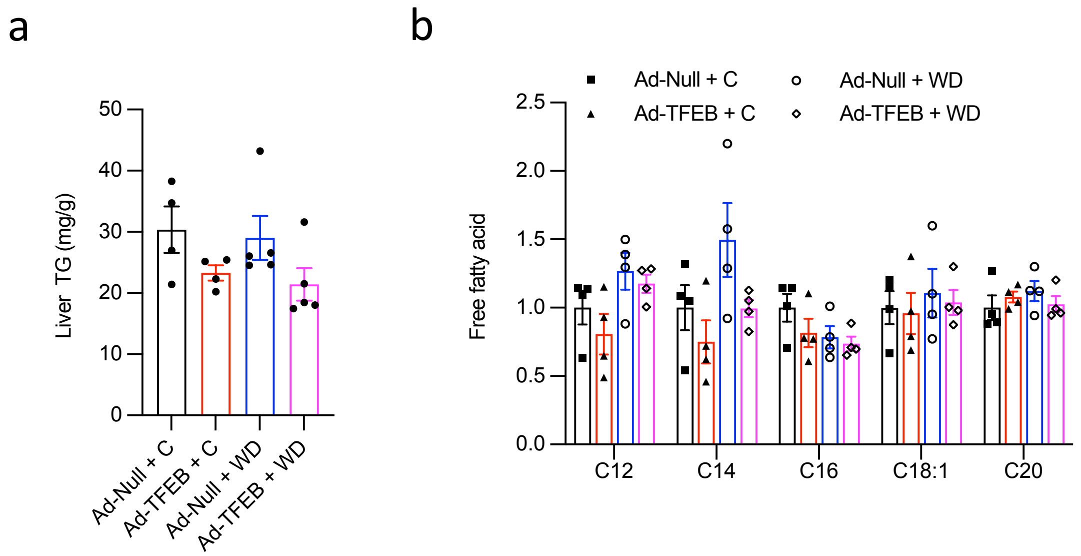

Supplementary Figure 5. Liver triglycerides and free fatty acids in liver TFEB overexpressing mice and controls. Male 10 weeks old C57BL/6J mice were intravenously injected with Ad-Null or Ad-TFEB at 5X10^8 pfu/mouse. One week later, mice were fed chow (C) or Western diet (WD) for one additional week. Mice were fasted for 6 h and euthanized. **a.** Liver triglycerides (TG) (n=4 for chow condition, n=5 for WD condition). **b.** Relative abundance of liver metabolites are shown with control arbitrarily set as 1 (n=4). All results are mean ± SEM. Statistical analysis for a was performed with 2-way ANOVA and Tukey post hoc test. Detailed statistical analysis of b is described under Metabolomics, statistical and bioinformatics analysis in the Methods section. Source data for a and b is provided as a Source Data file.

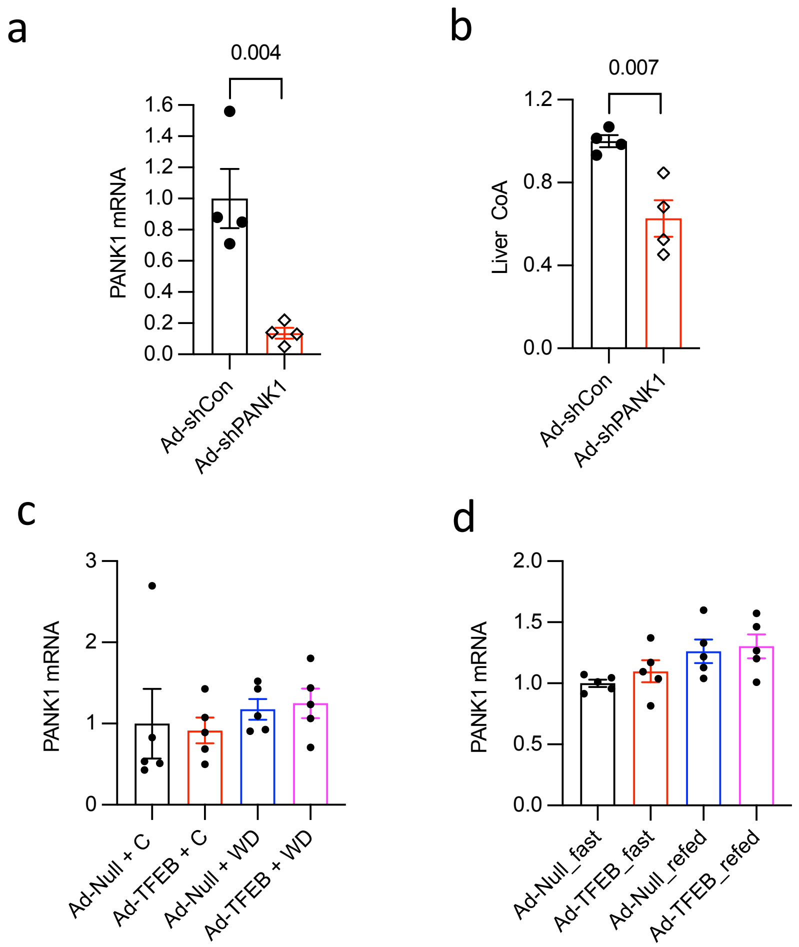

Supplementary Figure 6.TFEB does not induce hepatic PANK1 expression. a, b. Male 10 weeks old C57BL/6J mice were intravenously injected with Ad-shCon (Ad-scramble) or Ad-shPANK1 at 1X10^9 pfu/mouse. Mice were fed chow for 2 weeks and euthanized after 6 h fast (n=4). **a.** Liver patothenate kinase 1 (PANK1) mRNA. **b.** Liver coenzyme A (CoA) was measured by LC-MS method. **c.** Male 10 weeks old C57BL/6J mice were intravenously injected with Ad-Null or Ad-TFEB at 5X10^8 pfu/mouse. One week later, mice were fed chow (C) or Western diet (WD) for one additional week. Mice were fasted for 6 h and euthanized (n=5). **d.** Male 10 weeks old C57BL/6J mice were intravenously injected with Ad-Null or Ad-TFEB at 5X10^8 pfu/mouse. After 2 weeks, mice were either fasted for 22 h or fasted for 16 h and then refed with chow for 6 h (n=5). All results are mean ± SEM. Statistical analysis was performed with Unpaired 2-tailed t-test for a and b and 2-way ANOVA and Tukey post hoc test for c and d. A p value < 0.05 is considered statistically significant. Source data for a-d is provided as a Source Data file.

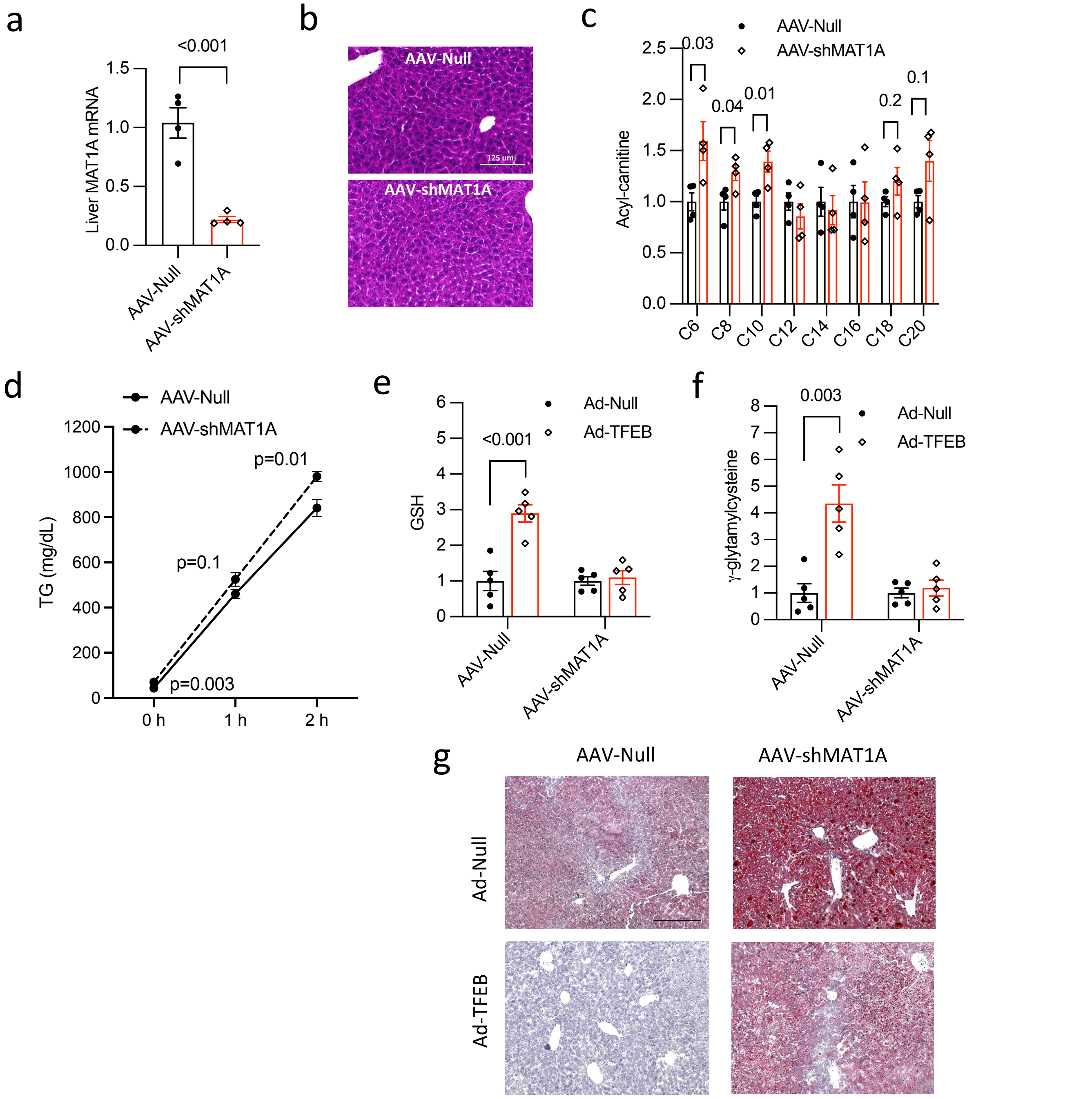

Supplementary Figure 7. Liver metabolites and VLDL-TG secretion in hepatic MAT1A deficient mice. a-c. Male 10 weeks old C57BL/6J mice were intravenously injected with AAV-Null or AAV-shMAT1A at 2X10[11] GC/mouse. Mice were maintained on chow diet for 2 weeks and euthanized after 6 h fast (n=4). **a.** Liver methionine adenosyltransferase 1A (MAT1A) mRNA. **b.** Representative liver H&E stain. Scale bar = 125 um. **c.** Relative abundance of liver acylcarnitines is shown with control arbitrarily set as 1. **d.** VLDL-TG secretion is measured in 10 weeks old male C57BL/6J mice injected with AAV-Null or AAV-shMAT1A at 2X10[11] GC/mouse and fed Western diet (WD) for 2 weeks. n=5 for AAV-Null; n=6 for AAV-shMAT1A. VLDL: very low-density lipoprotein; TG: triglyceride. **e, f, g.** Male 10 weeks old C57BL/6J mice were intravenously injected with AAV-Null or AAV-shMAT1A at 2X10[11] GC/mouse, and Ad-Null or Ad-TFEB (5X10[8] pfu/mouse) as indicated. Mice were then fed Western diet for 2 weeks and euthanized after 16 h fast (n=5). **e, f.** Relative abundance of liver reduced glutathione (GSH) and γ-glutamylcysteine is shown with respective control arbitrarily set as "1". **g.** Representative liver Oil Red O staining images are shown. Scale bar = 250 um. All results are mean ± SEM. A p value < 0.05 is considered statistically significant (Unpaired 2-tailed t-test for a, c, d, e, f). Source data for a, c, d, e, f is provided as a Source Data file. VLDL; very low-density lipoprotein; TG: triglyceride; MAT1A: methionine adenosyltransferase 1A.

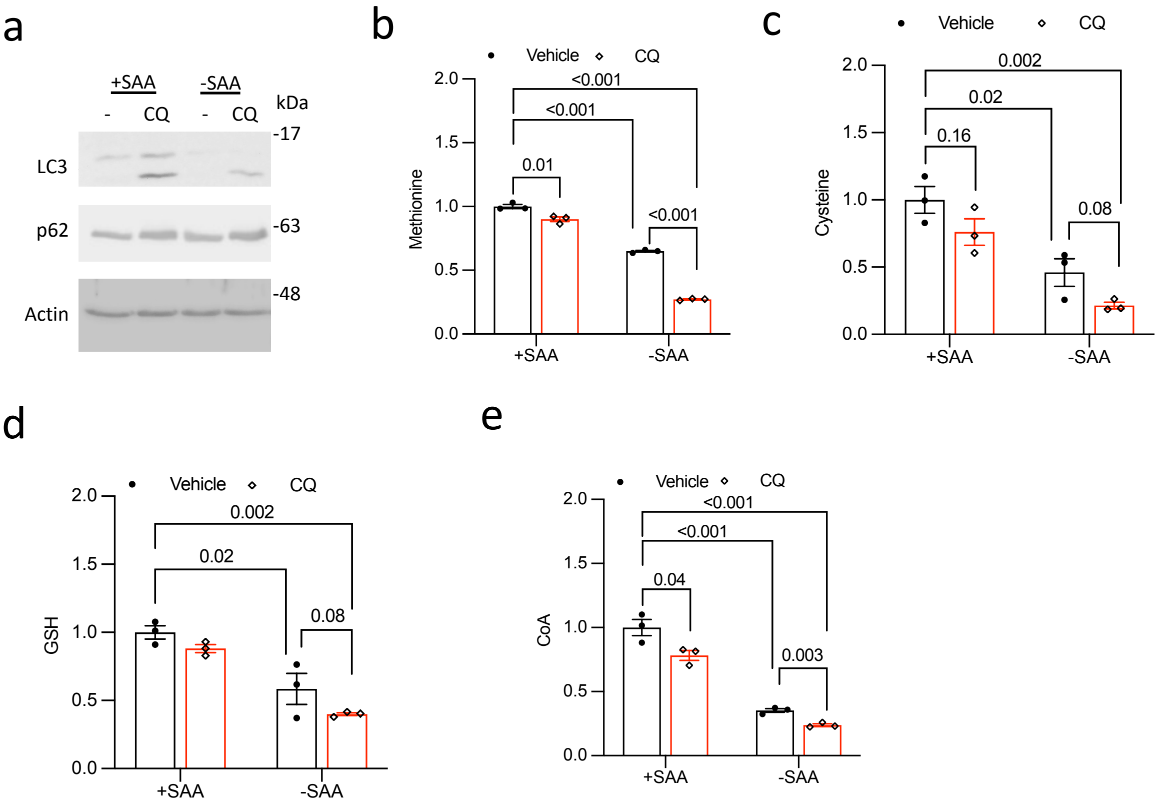

Supplementary Figure 8. Autophagy-lysosome pathway contributes to maintaining cellular cysteine and CoA homeostasis. AML12 cells were cultured in DMEM with ~200 µM methionine and cystine (+SAA) or methionine and cystine free DMEM (-SAA) for 6 h with or without chloroquine (CQ, 50 µM) as indicated. **a.** LC3 and p62 protein. Representative of 3 independent experiments. **b-e.** Metabolites in cell extracts were measured by LC-MS. Controls are arbitrarily set as 1. Results are expressed as mean ± SD of triplicate technical repeats of one experiment, which is representative of 2-3 independent treatments. A p value < 0.05 is considered statistically significant (unpaired 2-tailed t-test for b-e). Source data for a-e is provided as a Source Data file. SAA: sulfur amino acid; LC3: microtubule associated protein 1 light chain 3; p62: sequestosome 1; GSH: reduced glutathione; CoA: coenzyme A.

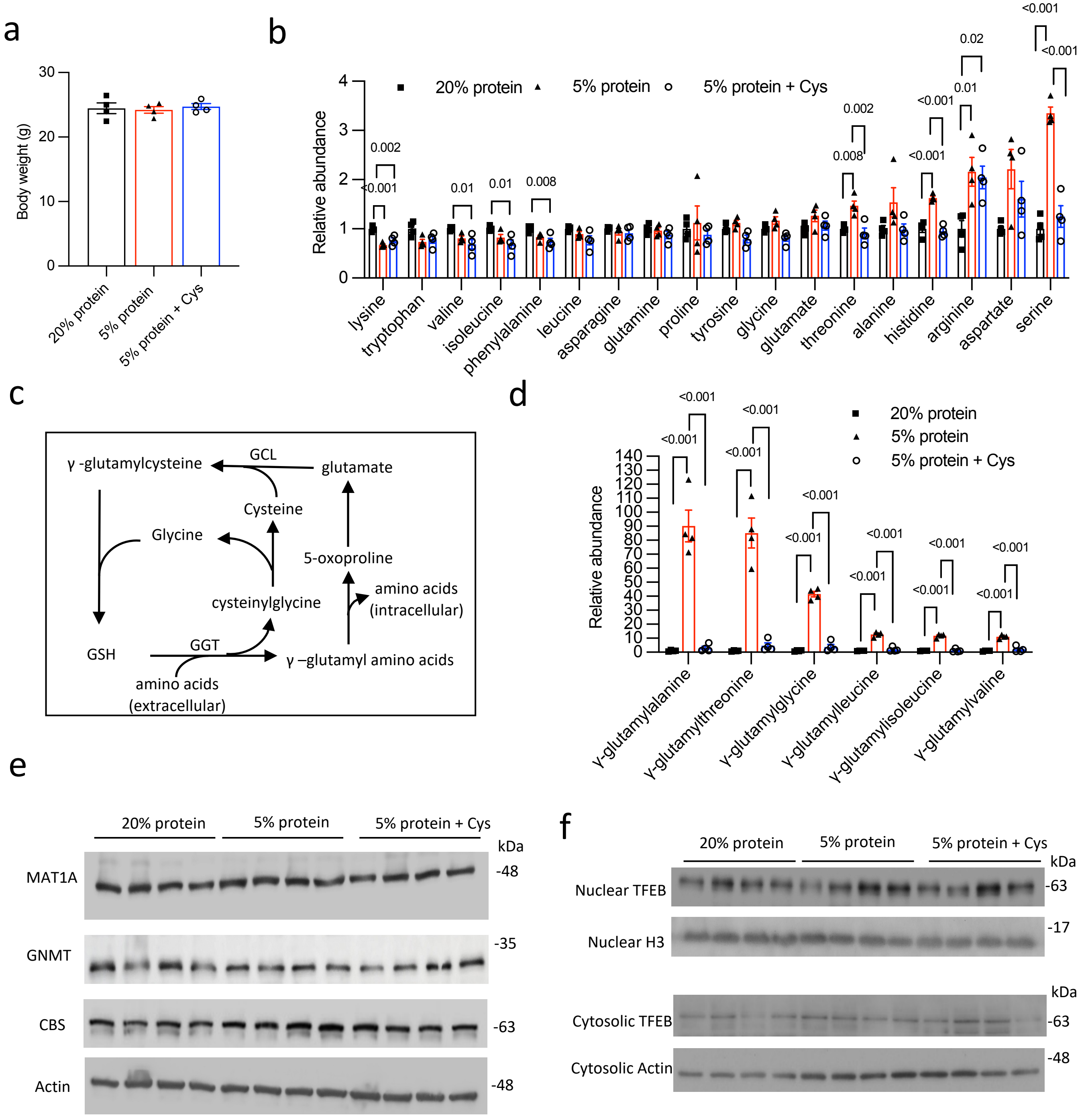

Supplementary Figure 9. Effects of hepatic cysteine deficiency on γ-glutamyl cycle and protein expression in mice. a, b, d, e, f. Male 10 weeks old C57BL/6J mice were fed isocaloric Western diet containing 20% protein, 5% protein or 5% protein with added cystine (Cys) for 6 weeks (n=4). Mice were euthanized after 6 h fast. **a.** body weight. (n=4). **b.** Liver tissues were used for metabolomics analysis (n=4). Relative abundance of liver metabolites are shown with control arbitrarily set as 1. Results are mean ± SEM. **c.** An illustration of the γ-glutamyl cycle. **d.** Liver tissues were used for metabolomics analysis (n=4). Relative abundance of liver metabolites are shown with control arbitrarily set as 1. Results are mean ± SEM. **e.** Western blot of protein in whole liver lysates. Each band represents an individual mouse sample. **f.** TFEB protein in liver nuclear and cytosolic fractions. H3: histone 3. Each band represents an individual mouse sample. A p value < 0.05 is considered statistically significant (One-way ANOVA and post hoc Dunnett test for a, b, d). Source data for a, b, d, e, f is provided as a Source Data file. GCL: Glutamate-Cysteine Ligase; GGT: γ-glutamyltransferase; GSH: reduced glutathione; MAT1A: methionine adenosyltransferase 1A; GNMT: glycine N-methyltransferase; CBS: Cystathionine β-Synthase; TFEB: transcription factor EB.

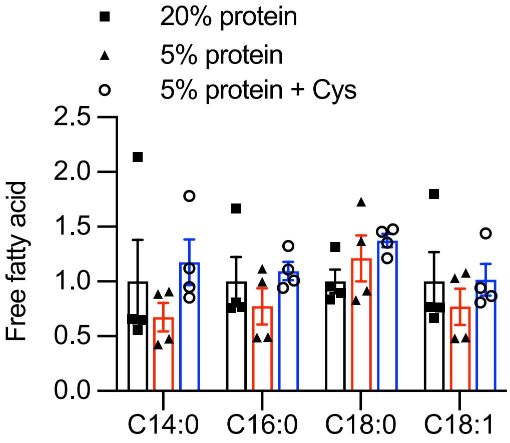

Supplementary Figure 10. Hepatic fatty acids in mice fed protein-adjusted diets. Male 10 weeks old C57BL/6J mice were fed isocaloric Western diet containing 20% protein, 5% protein or 5% protein with added cystine (Cys) for 6 weeks. Mice were euthanized after 6 h fast. Liver tissues were used for metabolomics analysis (n=4). Relative abundance of liver fatty acids are shown with control arbitrarily set as 1. All results are mean ± SEM. A p value < 0.05 is considered statistically significant (One-way ANOVA and post hoc Dunnett test). Source data is provided as a Source Data file.

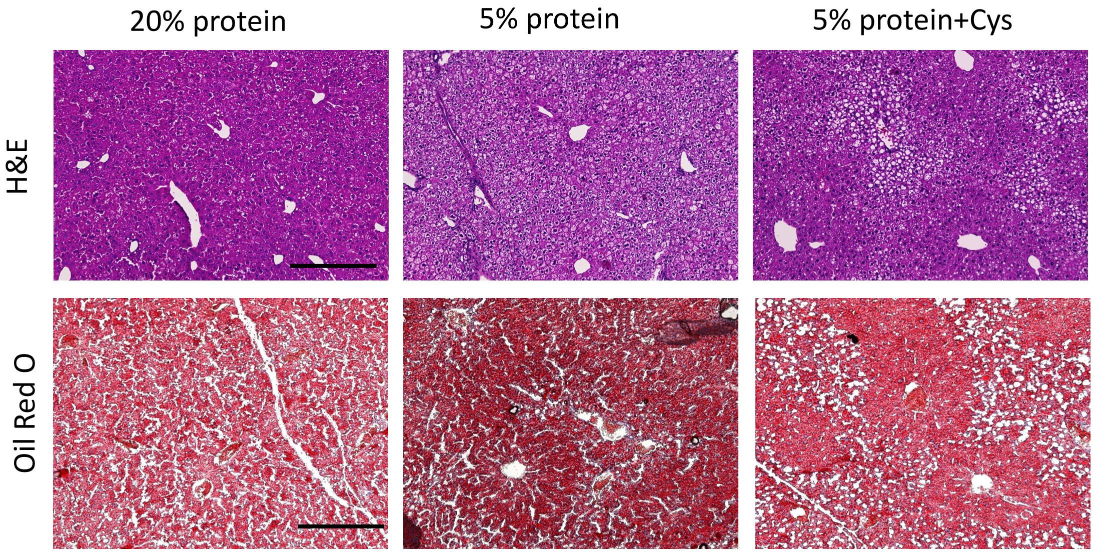

Supplementary Figure 11. Cystine supplementation partially reverse protein deficient WD-induced hepatic steatosis. Male 10 weeks old C57BL/6J mice were fed isocaloric Western diet containing 20% protein, 5% protein or 5% protein with added cystine (Cys) for 6 weeks. Representative liver H&E and Oil Red O stain are shown. Scale bar = 250 um. (n=4).

Supplementary Table 1. DNA oligos used for constructing PGL3 basic luciferase reporter plasmids containing putative TFEB binding sites that are underlined.

**Binding site (relative to transcription start site)**		**Sequence**
**H-1 (-59 bp)**	F	CTCTAGAggactcctccaattttctCACATGatttttcaggcactttcgA
	R	AGCTTcgaaagtgcctgaaaaatCATGTGagaaaattggaggagtccTCTAGAGGTAC
**H-2 (-153 bp)**	F	CTCTAGAtctttaccttaccttgacCAGGTGcttagagtttggaaagtcA
	R	AGCTTgactttccaaactctaagCACCTGgtcaaggtaaggtaaagaTCTAGAGGTAC
**H-3 (-399 bp)**	F	CTCTAGAaatgggaagagaccaatcCAGATGagacgcaggggaggagggA
	R	AGCTTccctcctcccctgcgtctCATCTGgattggtctcttcccattTCTAGAGGTAC
**H-4 (-451 bp)**	F	CTCTAGAcaaaaaaagctcagaataCAGGTGcgtgctcctgctctccctA
	R	AGCTTagggagagcaggagcacgCACCTGtattctgagctttttttgTCTAGAGGTAC
**H-5 (-467 bp)**	F	CTCTAGAatctgttgtgtaacatcaCAGCTGgctcagaataaaaaaacgA
	R	AGCTTcgtttttttattctgagcCAGCTGtgatgttacacaacagatTCTAGAGGTAC
**H-6 (-517 bp)**	F	CTCTAGAggtttctgttagcagaaaCACGTGgactcaaagcttttcctcA
	R	AGCTTgaggaaaagctttgagtcCACGTGtttctgctaacagaaaccTCTAGAGGTAC
**M-1 (-58 bp)**	F	CTCTAGAggcgagcccaatccagagCAGGTGtgggggtggagagaagccA
	R	AGCTTggcttctctccacccccaCACCTGctctggattgggctcgccTCTAGAGGTAC
**M-2 (-138 bp)**	F	CTCTAGAatctgttgtgtaatggtaCAGCTGgttcagggtacaggtgcaA
	R	AGCTTtgcacctgtaccctgaacCAGCTGtaccattacacaacagatTCTAGAGGTAC

Supplementary Table 2. Formulation of adjusted protein western diet. (Calculation based on that casein contains 0.25% cystine/cysteine and 2.3% methionine by weight)

	20% casein WD	5% casein WD	5% casein + Cys WD	WD (TD.88137, Envigo)
Added DL-Methionine (g/kg)	0	0	0	3
Added L-cystine (g/kg)	0	0	3.75	0
Casein (g/kg)	200	50	50	195
Sucrose (g/kg)	341.46	341.46	341.46	341.46
Corn Starch (g/kg)	100	250	246	150
Maltodextrin (g/kg)	47.9	46.75	46.75	0
Cellulose (g/kg)	50	50	50	50
Milk Fat (g/kg)	210	210	210	210
Cholesterol (g/kg)	1.5	1.5	1.5	1.5
Mineral Mix (g/kg)	35	35	35	35
Vitamin Mix (g/kg)	10	10	10	10
Total cysteine/cystine (%)	0.05	0.0125	0.3875	0.05
Total methionine (%)	0.46	0.115	0.115	0.76
Total sulfur amino acid (%)	0.5	0.1275	0.5	0.81
Protein (%)	17.4	4.4	4.7	17.3
Carbohydrate (%)	48.5	61.9	61.6	48.5
Fat (%)	21	21	21	21
Energy (Kcal/g)	4.5	4.5	4.5	4.5

Supplementary Table 3. Real-time PCR primer sequences. (The indicated location of the ChIP primers is relative to the transcription start site)

Name		Sequence
18S	F	GAGCGAAAGCATTTGCCAAG
	R	GGCATCGTTTATGGTCGGAA
ATG7	F	GTTCGCCCCCTTTAATAGTGC
	R	TGAACTCCAACGTCAAGCGG
ATG8	F	TTATAGAGCGATACAAGGGGGAG
	R	CGCCGTCTGATTATCTTGATGAG
ATP6V1H	F	GGATGCTGCTGTCCCAACTAA
	R	TCTCTTGCTTGTCCTCGGAAC
CTSB	F	TCCTTGATCCTTCTTTCTTGCC
	R	ACAGTGCCACACAGCTTCTTC
CSTD	F	GCTTCCGGTCTTTGACAACCT
	R	CACCAAGCATTAGTTCTCCTCC
LAMP1	F	CAGCACTCTTTGAGGTGAAAAAC
	R	ACGATCTGAGAACCATTCGCA
LIPA	F	TCTGGACCCTGCATTCTGAG
	R	CACTAGGGAATCCCCAGTAAGAG
TPP1	F	GAGTCTCACTTTTGCGCTGAA
	R	CTCCAGGGTTAGGTACTTTCCA
MAT1A	F	GTGCTGGATGCTCACCTCAAG
	R	CCACCCGCTGGTAATCAACC
CBS	F	CCAGGCACCTGTGGTCAAC
	R	GGTCTCGTGATTGGATCTGCT
CSE	F	TTCCTGCCTAGTTTCCAGCAT
	R	GGAAGTCCTGCTTAAATGTGGTG
PANK1	F	ATGGTAGACTGTAAAGGGTAC
	R	GGTTACCTCTTTGTGGGTGTC
h-MAT1A promoter (-479/-360)	F	TGAATCTGTTGTGTAACATCAC
	R	CCTTTGCTGTTGGGTGTCC
h-MAT1A promoter (-232/-126)	F	ACTACCAAGATTGGCTAAGAG
	R	CCAAACTCTAAGCACCTGGTC
h-MAT1A promoter (-82/-3)	F	CCCTCTTAGGAAATGGACTC
	R	GAGCGACTCCTATATATGGAA
m-MAT1A promoter (-507/-403)	F	CGGCAAAGATTTCCCTCGAAC
	R	CTACCTCCTCAGCGCAGAAC
m-MAT1A promoter (-467/-351)	F	AATGGTACAGCTGGTTCAGGG
	R	TGTGAAGGCTTCTCTCCACC

Supplementary Table 4. Description of Metabolon QC Samples.

Type	Description	Purpose
MTRX	Large pool of human plasma maintained by Metabolon that has been characterized extensively.	Assure that all aspects of the Metabolon process are operating within specifications.
CMTRX	Pool created by taking a small aliquot from every customer sample.	Assess the effect of a non-plasma matrix on the Metabolon process and distinguish biological variability from process variability.
PRCS	Aliquot of ultra-pure water	Process Blank used to assess the contribution to compound signals from the process.
SOLV	Aliquot of solvents used in extraction.	Solvent Blank used to segregate contamination sources in the extraction.

Supplementary Table 5. Metabolon QC Standards.

Type	Description	Purpose
RS	Recovery Standard	Assess variability and verify performance of extraction and instrumentation.
IS	Internal Standard	Assess variability and performance of instrument.

Original gel blots of Supplementary Figures

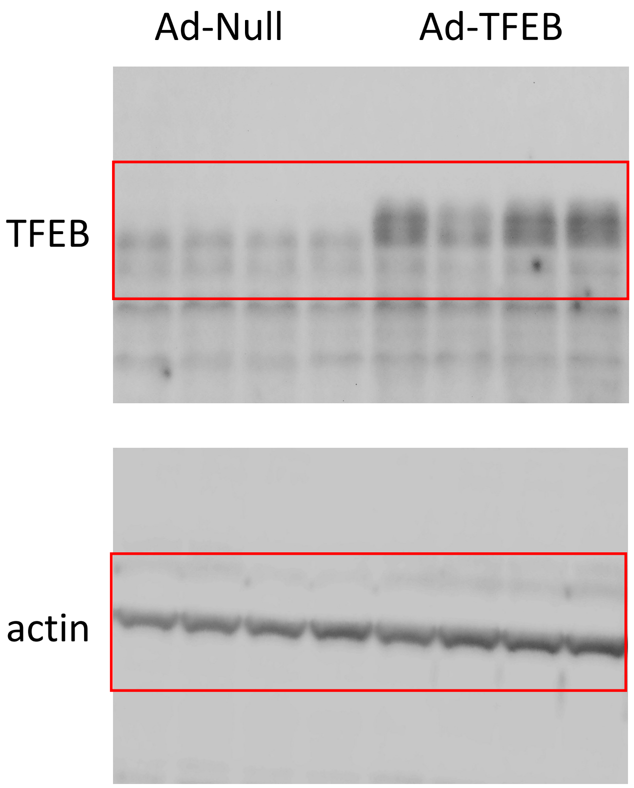

Supplementary figure 1c

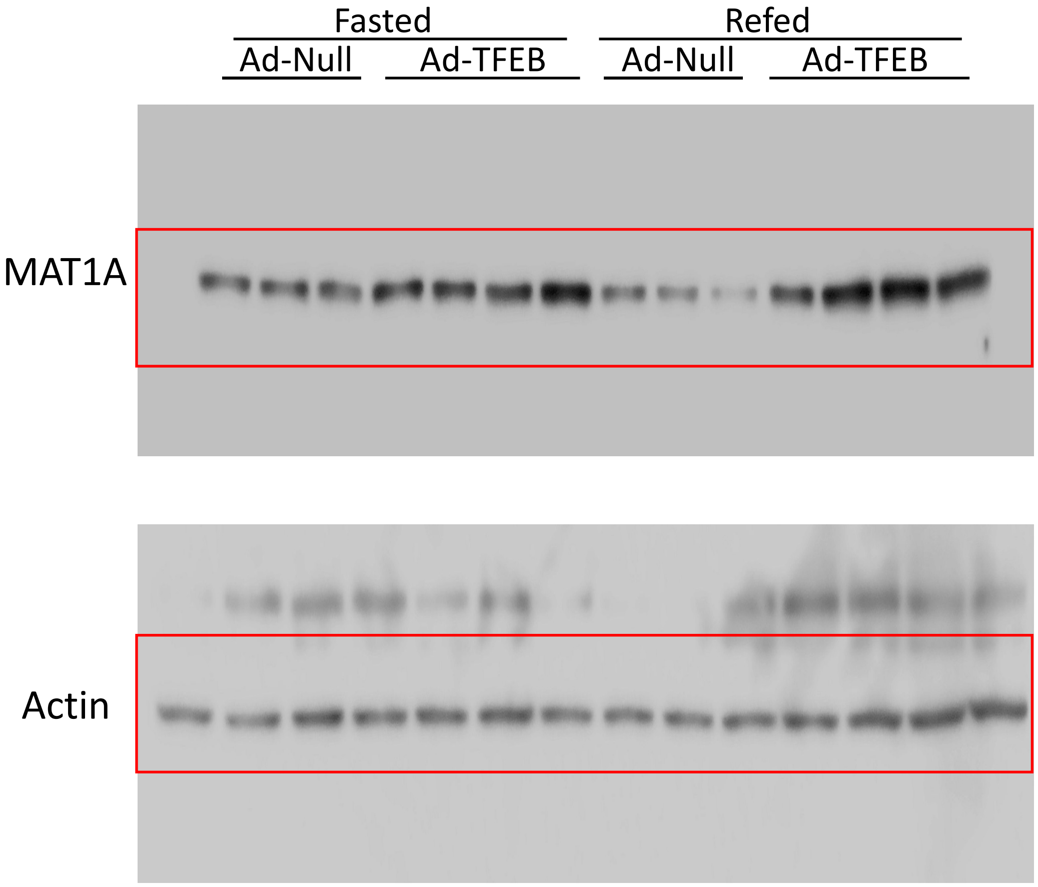

Supplementary figure 4a

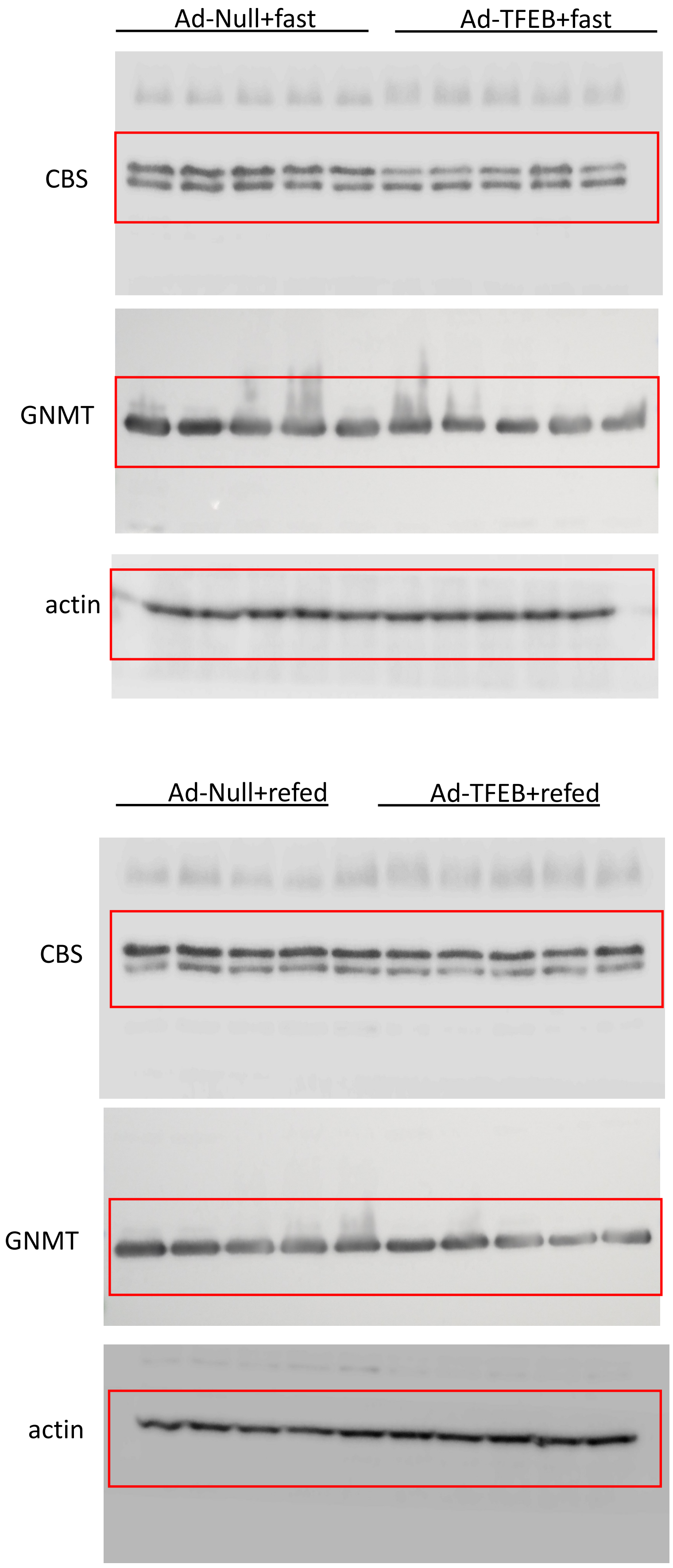

Supplementary figure 4e

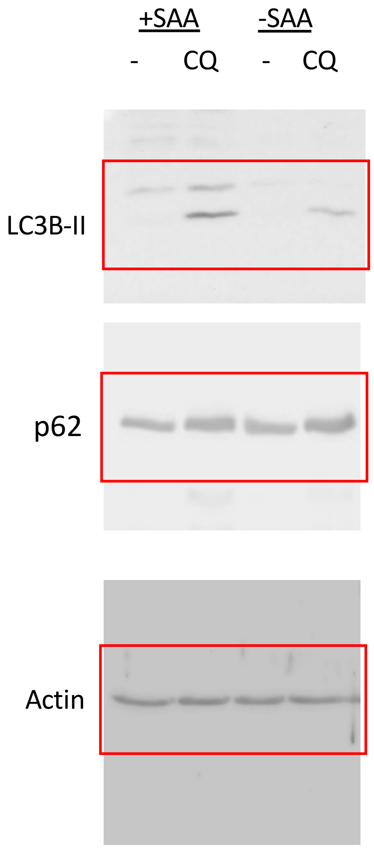

Supplementary figure 8a

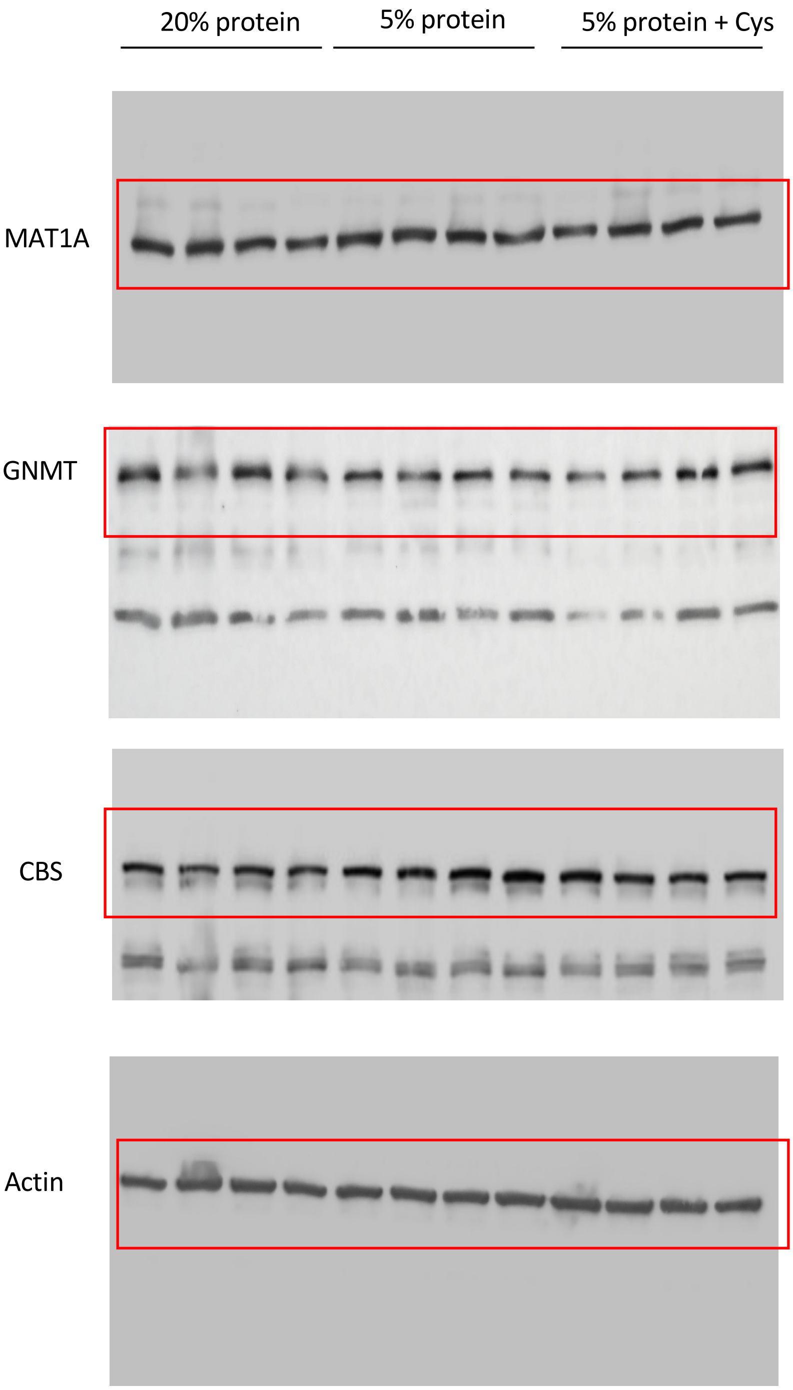

Supplementary figure 9e

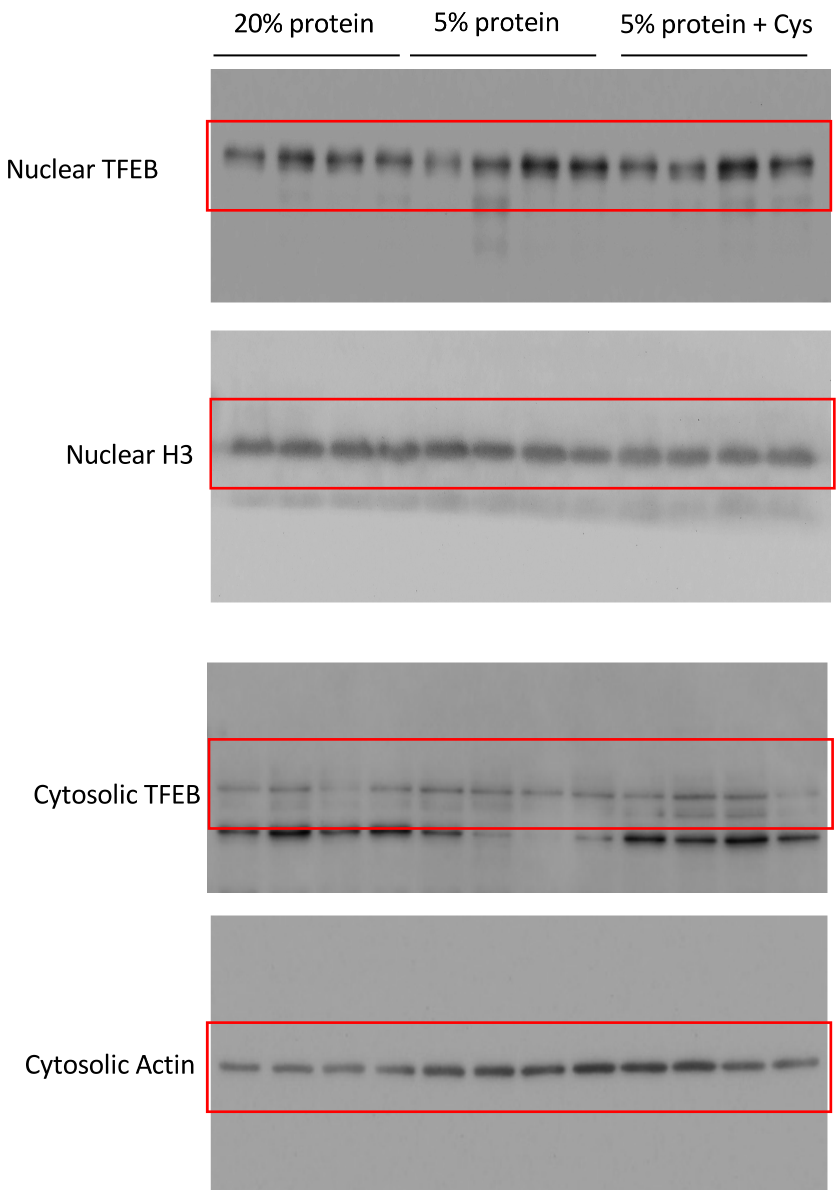

Supplementary figure 9f
