# Supplementary material for: TFEB regulates sulfur amino acid and coenzyme A metabolism to support hepatic metabolic adaptation and redox homeostasis
Source: Nat Commun. 2022 Sep 28;13:5696. doi: 10.1038/s41467-022-33465-9 (PMC9519740; doi:10.1038/s41467-022-33465-9)
Supplement: Supplementary file 3 — Reporting Summary [file 41467_2022_33465_MOESM3_ESM.pdf]

nature research

Corresponding author(s): Tiangang Li

Last updated by author(s): Aug 15, 2022

Reporting Summary

Nature Research wishes to improve the reproducibility of the work that we publish. This form provides structure for consistency and transparency in reporting. For further information on Nature Research policies, see Authors & Referees and the Editorial Policy Checklist .

Statistics

For all statistical analyses, confirm that the following items are present in the figure legend, table legend, main text, or Methods section.

n/a	Confirmed	
☐	☒	The exact sample size (*n*) for each experimental group/condition, given as a discrete number and unit of measurement
☐	☒	A statement on whether measurements were taken from distinct samples or whether the same sample was measured repeatedly
☐	☒	The statistical test(s) used AND whether they are one- or two-sided<br>*Only common tests should be described solely by name; describe more complex techniques in the Methods section.*
☒	☐	A description of all covariates tested
☐	☒	A description of any assumptions or corrections, such as tests of normality and adjustment for multiple comparisons
☐	☒	A full description of the statistical parameters including central tendency (e.g. means) or other basic estimates (e.g. regression coefficient) AND variation (e.g. standard deviation) or associated estimates of uncertainty (e.g. confidence intervals)
☐	☒	For null hypothesis testing, the test statistic (e.g. *F*, *t*, *r*) with confidence intervals, effect sizes, degrees of freedom and *P* value noted<br>*Give P values as exact values whenever suitable.*
☒	☐	For Bayesian analysis, information on the choice of priors and Markov chain Monte Carlo settings
☒	☐	For hierarchical and complex designs, identification of the appropriate level for tests and full reporting of outcomes
☒	☐	Estimates of effect sizes (e.g. Cohen's *d*, Pearson's *r*), indicating how they were calculated

Our web collection on statistics for biologists contains articles on many of the points above.

Software and code

Policy information about availability of computer code

Data collection	none
Data analysis	GraphPad Prism 6, R v3.5.1, MATLAB R2017a, ImageJ2

For manuscripts utilizing custom algorithms or software that are central to the research but not yet described in published literature, software must be made available to editors/reviewers. We strongly encourage code deposition in a community repository (e.g. GitHub). See the Nature Research guidelines for submitting code & software for further information.

Data

Policy information about availability of data

All manuscripts must include a data availability statement. This statement should provide the following information, where applicable:

- Accession codes, unique identifiers, or web links for publicly available datasets
- A list of figures that have associated raw data
- A description of any restrictions on data availability

The authors declare that all data supporting the findings of this study are available within the paper and its Supplementary Information files. Source data underlying all figures are provided as a Source Data file.

Field-specific reporting

Please select the one below that is the best fit for your research. If you are not sure, read the appropriate sections before making your selection.

☒ Life sciences ☐ Behavioural & social sciences ☐ Ecological, evolutionary & environmental sciences

Life sciences study design

All studies must disclose on these points even when the disclosure is negative.

Sample size	The sample size (n) of each experiment is provided in the corresponding figure captions in the main manuscript and supplementary information files. Sample sizes were chosen based on IACUC approved sample size that are expected to support meaningful conclusions but also minimize the number of animals needed for the study.
Data exclusions	no data exclusion
Replication	All in vitro experiments were replicated and reproducibility was confirmed for the number of repeated experiments stated in Figure legends. The sample size of each experiment involving mice is stated in the Figure Legends. These in vivo experiments were not repeated.
Randomization	Mice were ordered from the Jackson Lab. Upon arrival, they were assigned to each cage based on how they were divided in the crates. Each cage was randomly chosen to receive treatments. Cell culture experiments does not involve randomization because cells in different wells of the culture plates are from the same source.
Blinding	There was no blinding involved in the reported experiments because the sample conditions were known to the authors who performed the in vivo and in vitro experiments and data collection.

Reporting for specific materials, systems and methods

We require information from authors about some types of materials, experimental systems and methods used in many studies. Here, indicate whether each material, system or method listed is relevant to your study. If you are not sure if a list item applies to your research, read the appropriate section before selecting a response.

Materials & experimental systems

n/a	Involved in the study	
☐	☒	Antibodies
☐	☒	Eukaryotic cell lines
☒	☐	Palaeontology
☐	☒	Animals and other organisms
☒	☐	Human research participants
☒	☐	Clinical data

Methods

n/a	Involved in the study	
☒	☐	ChIP-seq
☒	☐	Flow cytometry
☒	☐	MRI-based neuroimaging

Antibodies

Antibodies used	Anti-TFEB antibody (A303-673A) was purchased from Bethyl Laboratoryies, Inc (Montgomery, TX). Actin antibody (ab3280), and CBS antibody (ab135626, Lot# GR3265732-15), CDO1 antibody (ab53436) were purchased from Abcam (Cambridge, MA). MAT1A antibody (# 712035, Lot# 2254811), GNMT antibody (PA5-76962, 1: 2000 dilution), P62 antibody (anti-SQSTM1, #89-015-843, 1:2000 dilution) were purchased from ThermoFisher Scientific (Waltham, MA). Antibodies against GCLC and GCLM were provided by Terry Kavanagh (University of Washington, Seattle, Washington, USA). LC3 antibody (Cat #. 3868S, 1:2000 dilution) was purchased from Cell Signaling Technology (Danvers, MA). Normal rabbit IgG (#2729, 1:50 dilution, Cell Signaling Technology, Danvers, MA)
Validation	TFEB, CDO1, MAT1A antibodies used in Western blotting were validated by overexpression and/or knockdown/knockout mouse liver samples. CBS and GNMT antibodies used in Western blotting to detect mouse target proteins were validated based on expected MW that was consistent with the on-line data published on the manufacturer's or vendor's website. GCLC and GCLM antibodies used in Western blotting to detect mouse target proteins were validated based on the expected MW of the target proteins that were consistent with the published study reporting the use of the same antibodies (PMID: 10593589). LC3 antibody and p62 antibody used in Western blotting to detect mouse target proteins were validated based on MW and the expected increase of LC3 and p62 protein upon the treatment of the autophagy inhibitor chloroquine.

Eukaryotic cell lines

Policy information about cell lines

Cell line source(s)	AML12 cells (originally from the American Type Culture Collection (ATCC CRL-2254)) were obtained from Yanqiao Zhang (Northeast Ohio Medical University, OH). HEK293A cells were purchased from Thermo Fisher Scientific (Grand Island, NY).
Authentication	No authentication was performed for AML12 cells. HEK293A cells were successfully used for amplification of replication defective adenovirus vectors.

Mycoplasma contamination	AML12 cells and HEK293A cells were not tested for micoplasma.
Commonly misidentified lines (See ICLAC register)	none used

Animals and other organisms

Policy information about studies involving animals; ARRIVE guidelines recommended for reporting animal research

Laboratory animals	Male, 10 weeks old C57BL/6J mice were ordered from the Jackson Lab. The sex and age were specified in the Figure Legends.
Wild animals	not used
Field-collected samples	not relevant
Ethics oversight	All animal protocols were approved by the Institutional Animal Care and Use Committee of the University of Oklahoma Health Sciences Center (#20-004) and the University of Kansas Medical Center (#2018-2457).

Note that full information on the approval of the study protocol must also be provided in the manuscript.
